# Exchanges

**Published:** 1869-02

**Authors:** 


### EXCHANGES.

We welcome first received in the list of exchanges with the IOWA JOURNAL; the American Journal Medical Sciences, edited by Isaac Hays, M. D., Philadelphia; the St. Louis Medical and Surgical Journal, edited by M. L. Linton, M. D., and G. Baumgarten, M. D.; the Boston Journal of Chemistry, edited by James R. Nicholds, M. D.; the Medical Record; the Medi- News and Library, published by H. C. Lea, Philadelphia; the Journal of Materia Medica, edited by Joseph Bates, M. D., and H. A. Tilden; the Pacific Medical and Surgical Journal, edited by Henry Gibbons, M, D., and assisted by Henry Gibbons, Jr., M. D., San Francisco; the St. Louis Medical Reporter, O. F. Patton, editor; the Medical and Surgical Reporter, a valuable weekly journal, S. W. Butler, M. D., and D. G. Benton, M. D., editors; the Humboldt Medical Archives, A. Hammer, M. D., and J. C. Whitehill, M. D. editors, St. Louis, Mo.; The Pharmacist, published by the Chicago College of Pharmacy; the Medical Investigator, T. C. Duncan, M. D., editor, Chicago; the Revista Medico, Quirurgica y Dentistica, redactores y proprietarios, Los Sres Wilson y Gonzales, Habana; Half Yearly Compendium of Medical Science, S. W. Butler, M. D., and D. G. Brinton, M. D., editors; the American Journal of Pharmacy, Wm. Proctor, Jr., editor; the Western Journal of Medicine, Theopholis Parvin, M. D, editor; the Dental Register, Cincinnati, R. J. Taft and C. Watt, editors; the Chemical News and Journal of Physical Science, edited by Wm. Crooks, F. R. S.; the American Naturalist, from the Peabody Academy of Science, Salem, Mass.; the Galaxy, from Sheldon & Co., New York.

To the foregoing editors and proprietors we express our hearty thanks, and trust they will pardon the delay in the issue of this number of our Journal.

